# Association of IL28B (IFNL3) rs12979860 mRNA levels, viral load, and liver function among HCV genotype 1a patients 

**Published:** 2019

**Authors:** Seyed Dawood Mousavi Nasab, Abbas Ahmadi Vasmehjani, Hooman Kaghazian, Rajab Mardani, Fatemeh Zali, Nayebali Ahmadi, Mohsen Norouzinia, Zahra Akbari

**Affiliations:** 1 *Department of Research and Development, Production and Research Complex, Pasteur Institute of Iran, Tehran, Iran*; 2 *Department of Microbiology and Immunology, Jahrom University of Medical Sciences, Jahrom, Iran *; 3 *Department of Virology, School of Public Health, Tehran University of Medical Sciences, Tehran, Iran*; 4 *Department of Biochemistry, Pasteur Institute of Iran, Tehran, Iran*; 5 *Department of Clinical Biochemistry, Faculty of Medicine, Tehran University of Medical Science, Tehran, Iran*; 6 *Proteomics Research Center, Faculty of Paramedical Sciences, Shahid Beheshti University of Medical Sciences, Tehran, Iran*; 7 *Gastroenterology and Liver Diseases Research Center, Research Institute for Gastroenterology and Liver Diseases, Shahid Beheshti University of Medical Sciences, Tehran, Iran*; 8 *Laser Application in Medical Sciences Research Center, Shahid Beheshti University of Medical Sciences, Tehran, Iran *

**Keywords:** HCV patients, Interleukin 28B, IFNL3, mRNA levels, Liver enzyme

## Abstract

**Aim:**

The present study was designed to evaluate the correlation of interleukin 28B (IL28B, IFNL3) rs12979860 mRNA levels, viral load, and liver function among hepatitis C virus (HCV) patients genotype 1a.

**Background:**

HCV is considered essentially hepatotropic and is a major health problem around the world.

**Methods:**

This study included 100 HCV-infected patients with HCV genotype1a (G1a) and rs12979860 CC genotype. These patients were divided into two groups according to HCV treatment. Aspartate aminotransferase (AST), alanine aminotransferase (ALT), alkaline phosphatase (ALP), and HCV Load were measured and recorded for each patient. IL28B mRNA levels were determined using real-time polymerase chain reaction assay, and their correlation with clinical data were analyzed. STRING was applied to construct a network and identify interactions between IL28B (*IFNL3*) and its significant neighbor proteins.

**Results:**

The results revealed a significant relationship between the ALT as well as ALP levels with IL28B rs12979860 mRNA expression level in men, and also with age >50 years. In the treated group, AST level and HCV load had a significant relationship with IL28B mRNA expression level. The results showed that the level of ALP and AST decreased significantly with increased IL28B mRNA expression level in the treated and untreated group, respectively. STRING database showed that IL28B (IFNL3) interacted with ten important neighbor proteins with some of these proteins being involved in signal transduction pathway activating antiviral response.

**Conclusion:**

This study indicated that rs12979860CC genotype could predict IL28B mRNA expression level in HCV-infected patients with G1a. Furthermore, IL28B mRNA expression level may serve as a useful marker for the development of G1a HCV-associated outcomes.

## Introduction

 Hepatitis C virus (HCV) is a major health problem around the world. HCV infection has acute and chronic stages, where two distinct results from acute infection; HCV is spontaneously removed from the body of at least 20% of the patients, indicating a strong immune mechanism against HCV (1, 2). In the remaining cases, chronic infections develop, of which half of the cases respond to treatment with standard treatment, PEGylated-IFNα/Ribavirin, where 80% of the infected people have genotypes 2 and 3 of the virus and 50% of the infected genotype1a (G1a) treatment (3, 4). Therefore, understanding the controversy of contaminated hosts against HCV infection is clinically helpful. 

Several factors have been identified in determining the outcomes of the disease during the normal course of HCV infection which provides a good prognosis for the disease. The viral factors involved can be virus genotype, viral load, and genetic variants of the virus itself, with host factors including age, gender, host genetics (5). In this case, host immunological genetic factors are very important. Interferons have an important role in HCV infection, such that they are used as a good measure of response to treatment in these individuals. Interferon lambda (IFNL), which expresses IL28B (IFNL3), plays an important role in viral infections. This cytokine has 3 genotypes, including CC, TT, and CT, which are found in a wide variety of populations worldwide (6). Genome-wide association studies (GWAS) have shown that the cytokine variants of polymorphism, in particular IL28B (IFNL3) rs2979860, is associated with host defense against HCV (7-9). Furthermore, a correlation has been reported between viral load and some interleukins, which is also affected by different HCV genotypes (10, 11).

Type III interferons (IFNs), also termed IFN-λ, are important players in immunity to viral and bacterial infections. IFNL3 (IFN-λ3/IL28B) along with IFNL1 (IFN-λ1/IL29) and IFNL2 (IFN-λ2/IL28A) belong to the interferon-λ (IFNL) cytokine family (12, 13). 

In Iran, only the frequency of CC allele has been reported as a good treatment allele as well as its relationship with the results of the treatment response (6, 14, 15). However, limited studies have been conducted on the effect of IL28B variants on HCV infection and IL28B production mRNA expression levels. Therefore, the IL28B polymorphism may be a factor indicating the resistance or susceptibility of the treatment to the infection. The aim of this study was to evaluate the association of IL28B (IFNL3) rs12979860 mRNA levels, viral load, and biochemical parameters (ALT, AST, ALP) in Iranian HCV patients infected with HCV G1a. 

## Methods

**Study design and population**

Blood samples of confirmed patients with chronic hepatitis C were collected with inclusion criteria registered at the Honary Medical Clinic Centre in Jahrom city of Shiraz, Iran, from January 2018 to December 2018. The HCV genotyping was determined as described previously (16), and G1a HCV patients were studied in this work. These patients were divided into two groups according to PEGylated-IFNα/Ribavirin treatment/and un-treatment HCV treatment. Patients were examined in terms of age (20-68 years), gender (man and woman), and weight (55-80kg). The HCV RNA levels were measured using the Gene Proof Hepatitis C Virus (HCV) PCR Kit (Gene Proof, Brno, Czech Republic).

**IL28B (IFNL3) genotyping by RFLP**

Genomic DNA was extracted using the QIAamp DNA Blood Mini Kit (Qiagen, Hilden, Germany). The IL28B rs12979860 SNPs were genotyped by the PCR-RFLP method as described by Mousavi Nasab and colleagues (6). 

**Clinical chemistry**

*Alanine aminotransferase (*ALT),* aspartate transaminase* (AST) and alkaline phosphatase (ALP) activity was determined by the colorimetric method using Olympus AU400 auto-analyzer machine (Mishima Olympus Co. Ltd., Shizuoka-ken, Japan) in the plasma samples. Reference values for ALT, AST, and ALP were set at (7 to 55), (8 to 48), and (36-113), respectively, and data were reported as international units (IU)/L.

*IL28B* (*IFNL3*)* mRNA levels by Real-Time PCR assay*

Isolation of peripheral blood mononuclear cells (PBMCs) of patient samples was performed using Ficoll method, (FicollPaque plus GE Healthcare). Then, RNA was extracted using TRIzol according to the manufacturer's protocol. Afterward, the concentration and quality of each sample were measured with the NanoDrop (Thermo fisher scientific). The expression of IL28B mRNA level was evaluated using real-time PCR, as previously described (15). Briefly, the total RNAs (1 µg) were extracted using the TRIzol reagent (Invitrogen, USA), followed by cDNA synthesis kit (Takara, Japan). Real-time PCR using SYBR Green supermix (Amplicon, Tehran, Iran) and the IL28B mRNA primers was performed by real-time PCR machine (ABI step one plus, Applied Biosystems, USA). Finally, the expression level was determined using the equation 2^−ΔΔCt^.

**Interaction network construction**

We applied the Search Tool for the Retrieval of Interacting Genes/Proteins (STRING) (17) to construct a network and identify interactions between IL28B (*IFNL3*) and its significant neighbors. The interactions include direct (physical) and indirect (functional) associations.

**Statistical analysis**

SPSS version 20 software (SPSS Inc., Chicago, IL, USA) was used for statistical analyses including the basic descriptive and frequency features. The relationship between two variables was tested by Spearman. The relationship between the level of IL28B rs12979860 mRNA expression and independent variables was captured using logistic regression. P-values less than 0.05 were considered to be statistically significant. 

## Results

A total of 100 HCV patients with G1a and rs12979860 CC genotype were included in the study. Clinical and laboratory parameters of patients are summarized in Table 1. The results showed that, unlike women, there is a significant relationship between the ALT as well as ALP levels and the IL28B (*IFNL3*) mRNA expression level in men (Table 2). There is also a significant relationship between ALT as well as ALP levels and IL28B mRNA expression level in people aged 50-68 years (Table 3; p =0.02, p=0.04). In the treated group, AST level has a significant relationship with IL28B mRNA expression level (Table 4, p = 0.02). Also, there was a significant relationship between IL28B mRNA expression level and viral load in the treated group (p = 0.04), but no difference was observed in the untreated group. Regression results in Table 5 showed that the level of AST decreased significantly (3.51) per unit with increase in the amount of IL28B mRNA expression level in the treated group. The regression results showed that ALP level diminished by 22.4 units per unit increase in IL28B mRNA expression level in the untreated group (Table 5).

**Figure 1:**
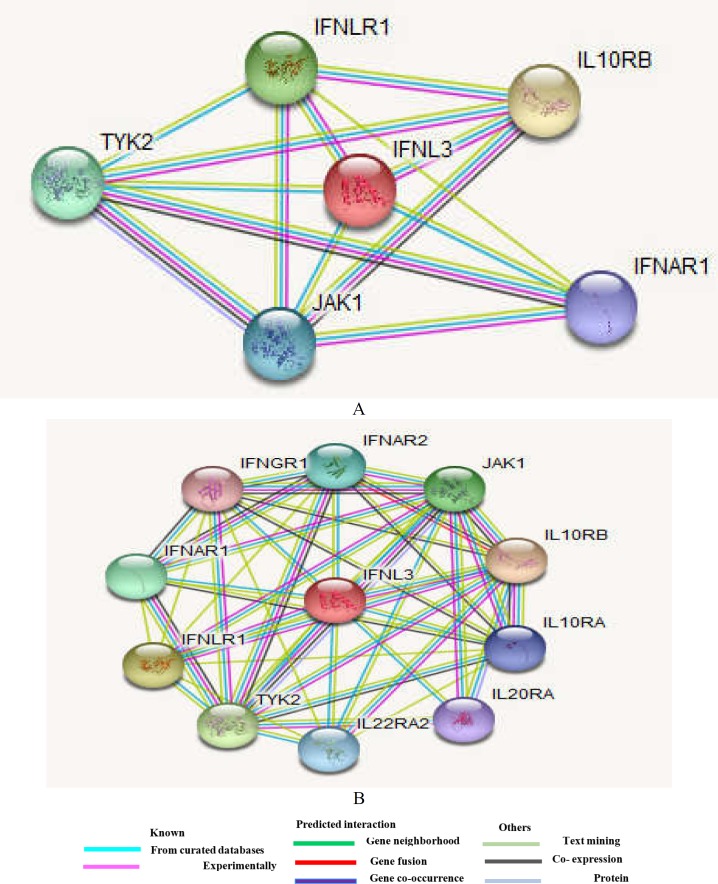
A) IL28B (IFNL3) and ten first neighbors (11 nodes and 46 edges). B) IL28B (IFNL3) and five closest neighbors (6 nodes and 14edges) (https://string-db.org/cgi/network). IFN: Interferon; IL: Interleukin; R: Receptor; A: alpha; B: beta; JAK: Janus kinase; Tyk2: Tyrosine kinase 2

Interactions between IL28B (*IFNL3*) and ten important neighbors were obtained from STRING database, as presented in Figure 1. Among them, the first five proteins (*IL10RB, IFNLR1, TYK2, TYK1*, and *IFNAR1*) are involved in signal transduction pathway activating the antiviral response.

**Table 1:** Clinical and laboratory parameters in hepatitis C patients

Variables	Treated (n=50)	Un-Treated (n=50)	P value
Sex:			0.36
Male	27 (57%)	23 (43%)
Female	23 (43%)	27 (57%)
Liver enzyme^a^:			
ALT (IU/L)	58.2±2.1	68.2±5.5	0.08
AST (IU/L)	37.80±1.19	41.4±4.2	0.15
ALP (IU/L)	245±12.5	245±12.5	0.38
Viral Load (IU/mL)^a^	-	1.4837×10^6^±9.0×10^4^	
Phase of Disease:			0.43
Acute	0 (0%)	23 (56%)	
Chronic	28 (100%)	27 (44%)	
IL28 mRNA level^a^	0.17± 0.057	0.05±0.049	0.03

^a ^Data was expressed as Mean ± SD for quantitative measures and both number and percentage for categorized data. *ALT: Alanine aminotransferase*, *AST: aspartate transaminase*, ALP: alkaline phosphatase, IL28B: interleukin-28B

**Table 2:** Correlation of IL28B (*IFNL3*) mRNA and viral load levels with liver enzymes based on gender in patients infected with HCV genotype 1a

Gender		Variable	AST r value P-value	ALT r value P-value	ALP r value P-value
Male	IL28 mRNA level		-0.11 0.44	-0.42 0.002	-0.35 0.01
	HCV Load		-0.08 0.70	0.07 0.74	0.03 0.88
Female	IL28 mRNA level		-0.05 0.84	-0.32 0.18	-0.19 0.39
	HCV Load		0.1 0.75	0.51 0.08	0.40 0.18

*ALT: Alanine aminotransferase*, *AST: aspartate transaminase*, ALP: alkaline phosphatase, HCV: Hepatitis C virus, IL28B: interleukin-28B

**Table 3:** Correlation of IL28B mRNA and viral load levels with liver enzymes according to age groups in patients infected with HCV genotype 1a

Age		Variable	AST r value P-value	ALT r value P-value	ALP r value P-value
20-35	IL28 mRNA level		0.23 0.32	-0.39 0.07	-0.24 0.28
	HCV Load		-0.13 0.65	0.19 0.52	0.33 0.25
35-50	IL28 mRNA level		-0.13 0.48	-0.31 0.09	-0.25 0.17
	HCV Load		0.08 0.77	0.21 0.44	0.03 0.91
50-68	IL28 mRNA level		-0.32 0.18	-0.53 0.02	-0.47 0.04
	HCV Load		-0.58 0.08	0.07 0.84	0.07 0.85

ALT: Alanine aminotransferase, AST: aspartate transaminase, ALP: alkaline phosphatase, HCV: Hepatitis C virus, IL28B: interleukin-28B

**Table 4:** Correlation of IL-28B mRNA and viral load levels with liver enzyme based on treatment in patients infected with HCV genotype 1a

HCV therapy	Variable	AST r value P-value	ALT r value P-value	ALP r value P-value
Treated	IL28 mRNA level	-0.43 0.02	-0.18 0.48	-0.22 0.25
	HCV Load	-0.04 0.81	0.16 0.41	0.21 0.27
Un-Treated	IL28 mRNA level	-0.26 0.47	-0.25 0.48	0.005 0.98
	HCV Load	0.07 0.84	0.21 0.56	-0.12 0.75

*ALT: Alanine aminotransferase*, *AST: aspartate transaminase*, ALP: alkaline phosphatase**,** HCV: Hepatitis C virus, IL-28B: interleukin-28B

**Table 5:** Regression analysis of rs12979860 IL-28B mRNA level with *aspartate transaminase* (AST), *alanine aminotransferase* (ALT), and alkaline phosphatase (ALP) based on treatment in patients infected with HCV genotype 1a

HCV therapy	Variable	B (SE)	p-value
Treated	IL28 mRNA level	-22.44(13.43)	0.11
	ALP	-50.27(33.29)	0.14
Un-Treated	IL28 mRNA level	-13.02(13.42)	0.38
	ALP	-142.72(55.09)	0.05
Treated	IL28 mRNA level	-3.51(1.27)	0.01
	AST	-4.64(3.15)	0.15
Un-Treated	IL28 mRNA level	-0.27(1.19)	0.83
	AST	1.78(4.92)	0.73
Treated	IL28 mRNA level ALT	0.47(0.5) 0.45(2.32)	0.65 0.45
Un-Treated	IL28 mRNA level ALT	0.65(0.5)	0.35
		0.4(0.75)	0.6

B (SE): beta (unstandardized coefficients), HCV: Hepatitis C virus, IL-28B: interleukin-28B

## Discussion

Interferon lambda-3 (*IFNL3*), also termed IL28B, has antiviral activity in addition to immune system activity (18, 19). 

The studies indicate that rs12979860 polymorphism of IL28B(*IFNL3*) gene is significantly related to the results of HCV infection (8, 20). The causative effect of rs12979860 polymorphism on host protection against HCV infection is known, but it has been suggested that this polymorphism may have an effect on the expression and production of cytokine protein in the course of the infection and on disease clinical outcomes. 

Previous studies revealed that IL28B (*IFNL3*) is associated with outcomes of chronic HCV infection. Our results showed that liver enzyme levels correlated with a high level of rs12979860 IL28B (*IFNL3*) mRNA expression. Hence, in our findings, a significant difference was observed between rs12979860 IL28B mRNA expression level and ALT, AST levels, and viral load. Similar results were reported by Hendy et al. (21). 

Incongruent with our study, Khairy et al. (22) showed no significant difference between IL28B genotypes regarding pretreatment ALT and AST levels and viral load. 

In a study, it was observed that individuals with IL28B CC genotype on rs12979860 had a lower AST level and better liver function recovery (23). Another study reported that the expression levels of IL28B were lower in PEG-IFN-treated patients with rs8099917 genotype (24). Elsewhere, rs8099917 was the only SNP that significantly correlated with the IL28B serum levels, while the other SNPs failed to show any correlation with IL28B levels (25). However, further investigations are required to explain the effect of IL28B mRNA expression levels on liver function and its role in treatment predictor. A plausible explanation of increased expression of IL28B levels in patients undergoing treatment could be the unique capability of IFN-λs to enhance its expression when induced by IFN-α; that is, patients under treatment with PEG-IFN α/β-ribavirin would most likely have elevated levels of IL28B (*IFNL3*) levels in response to its direct stimulation with IFN-α (26).

Our results showed that rs12979860IL28B (*IFNL3*) variant has an effect on the production of IL28B, which is associated with therapy response. The CC genotype of rs12979860 was the most prevalent genotype of IL28B gene among HCV-infected patients and it was associated with higher IL28B serum levels (11). Further investigations are required to understand how the IL28B levels vary according to the IL28B (*IFNL3*) genetic polymorphisms, since such polymorphisms could affect the expression and stability of IL28B mRNA. Also, it is not clear at what level the expression of IL28B is affected in HCV-infected patients undergoing treatment with PEGylated-IFNα/Ribavirin.

IFNL3 acts as a ligand for the heterodimeric class II cytokine receptor composed of Interleukin-10 receptor subunit beta (*IL10RB*) and Interferon lambda receptor 1 (*IFNLR1*). This receptor engagement leads to the activation of the Janus kinase and signal transducer and activator of transcription (JAK/STAT) signaling pathway resulting in the expression of IFN- stimulated genes (ISG), which are required to control viral infection. The rs12979860 polymorphism, with its particular location upstream of the promoter region of the *IFNL3* gene as well as of the *IFNL1* and *IFNL2* genes, can theoretically influence all three IFN-lambda genes. From the STRING database, we found that IL28B interacted with ten important neighbor proteins. Among them, five first proteins (*IL10RB, IFNLR1, TYK2, TYK1,* and *IFNAR1*) are involved in signal transduction pathway activating the antiviral response (12, 13, 27). 

This study indicated that variation at SNP rs12979860 could predict IL28B (*IFNL3*) mRNA expression level in HCV-infected patients with genotype 1a. Furthermore, IL28B mRNA expression level may serve as a useful marker for the development of HCV-associated outcomes. IL28B rs12979860 polymorphisms may be associated with mRNA expression and correlated with host protection against HCV infection regarding viral load and HCV genotype.
